# RecG Directs DNA Synthesis during Double-Strand Break Repair

**DOI:** 10.1371/journal.pgen.1005799

**Published:** 2016-02-12

**Authors:** Benura Azeroglu, Julia S. P. Mawer, Charlotte A. Cockram, Martin A. White, A. M. Mahedi Hasan, Milana Filatenkova, David R. F. Leach

**Affiliations:** Institute of Cell Biology, School of Biological Sciences, University of Edinburgh, Edinburgh, United Kingdom; Oregon State University, UNITED STATES

## Abstract

Homologous recombination provides a mechanism of DNA double-strand break repair (DSBR) that requires an intact, homologous template for DNA synthesis. When DNA synthesis associated with DSBR is convergent, the broken DNA strands are replaced and repair is accurate. However, if divergent DNA synthesis is established, over-replication of flanking DNA may occur with deleterious consequences. The RecG protein of *Escherichia coli* is a helicase and translocase that can re-model 3-way and 4-way DNA structures such as replication forks and Holliday junctions. However, the primary role of RecG in live cells has remained elusive. Here we show that, in the absence of RecG, attempted DSBR is accompanied by divergent DNA replication at the site of an induced chromosomal DNA double-strand break. Furthermore, DNA double-stand ends are generated in a *recG* mutant at sites known to block replication forks. These double-strand ends, also trigger DSBR and the divergent DNA replication characteristic of this mutant, which can explain over-replication of the terminus region of the chromosome. The loss of DNA associated with unwinding joint molecules previously observed in the absence of RuvAB and RecG, is suppressed by a helicase deficient PriA mutation (*priA300*), arguing that the action of RecG ensures that PriA is bound correctly on D-loops to direct DNA replication rather than to unwind joint molecules. This has led us to put forward a revised model of homologous recombination in which the re-modelling of branched intermediates by RecG plays a fundamental role in directing DNA synthesis and thus maintaining genomic stability.

## Author Summary

DNA double-strand breaks are accurately repaired by homologous recombination. This accuracy is ensured by copying the correct genetic information present on a second unbroken copy of the DNA, normally a sister chromosome that is generated during DNA replication. This implies that DNA synthesis occurring during recombination must be directed to replace lost or damaged base pairs but not to over-replicate undamaged chromosomal regions. Here, we investigate the genomic consequences of the absence of RecG during DNA repair following a site-specific double-strand break introduced in only one of two homologous *E*. *coli* chromosomes. Our observations suggest that RecG can re-model branched intermediates of recombination to direct the correct binding of PriA. This establishes converging replication forks that replace lost DNA at the site of DSBR and prevents over-replication of flanking DNA regions. This has led us to re-evaluate our understanding of the pathway of homologous recombination in *E*. *coli* and to propose a model in which RecG plays a critical role in remodelling branched intermediates at the interface of recombination and DNA replication.

## Introduction

**Fig 1:**
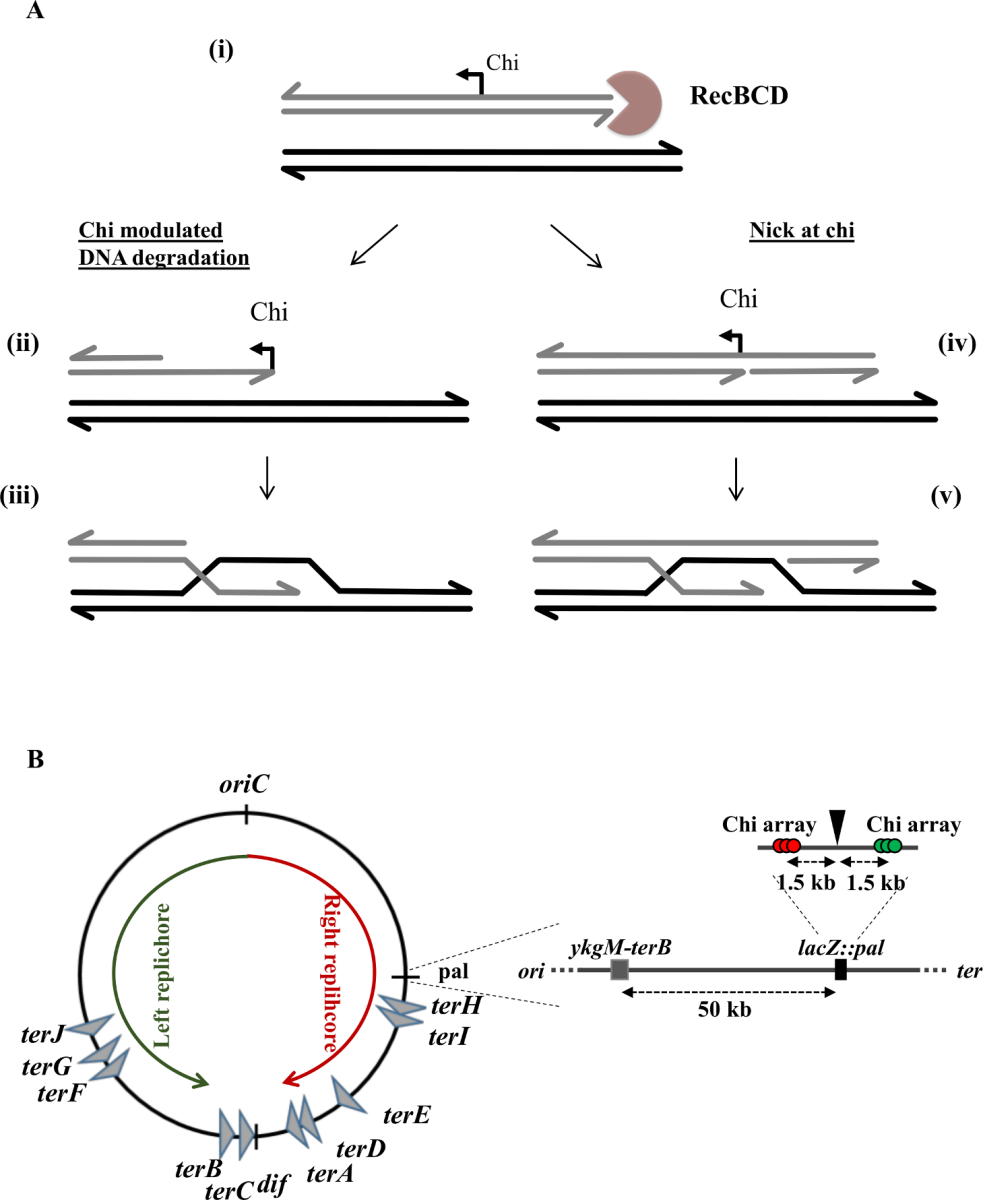
Current models for RecBCD action and Chi cleavage in the initiation of recombination and schematic depiction of the site of DSBR used in this work. A. Current alternative hypotheses for the initiation of recombination by RecBCD [2,3]. RecBCD (pink figure) loads at the site of a DSB and translocates along the duplex DNA (i). During translocation, RecBCD either degrades both strands up to the recognition of a correctly oriented Chi site (with a preference for cleaving the 3’ terminal strand) or unwinds the duplex DNA without degrading it. Once a correctly oriented Chi site is recognised, the complex undergoes a conformational change and either up-regulates 5’ to 3’ cleavage while inhibiting 3’ to 5’ cleavage (ii) or nicks the 3’ terminal DNA strand and continues unwinding (**iv**). Both of these scenarios “Chi modulated DNA degradation” and “nick at Chi” lead to the formation ssDNA with a 3’ terminus, which is a substrate for the loading and polymerisation of RecA. These alternative hypotheses for the initiation of recombination lead to the formation of different structures of joint molecules (iii and v) and therefore to different biochemical steps following strand invasion and D-loop formation by RecA coated DNA. B. Map of the *E*. *coli* chromosomal depicting the two replichores and the site of DSBR used in this work. The chromosome of *E*. *coli* is drawn as a black line and the directions of replication of the left and right replichores are indicated by green and red arrows respectively. The regions of DSB induction in *lacZ* and of insertion of an ectopic *terB* site in *ykgM-terB* are shown in more detail. The palindrome and Chi arrays are shown by a black triangle and three coloured circles, respectively.

In wild type *Escherichia coli* cells, DNA double-strand break repair (DSBR) is mediated by the RecBCD pathway of homologous recombination. In this pathway, DNA is unwound by RecBCD and cleaved five nucleotides 3’ of the sequence known as Chi (5’-GCTGGTGG-3’) [1]. Following recognition of Chi, RecBCD continues to unwind the substrate and facilitates the loading of RecA onto the 3’ strand close to Chi. *In vitro*, the fates of the DNA strands between the DNA double-strand break (DSB) site and Chi and of the strand terminating 5’ at Chi depend on the ATP/Mg^2+^ concentration ratio. Degradation of these strands increases *in vitro* as the ATP/Mg^2+^ concentration ratio increases but the extent of degradation *in vivo* is unknown. Two recent reviews of the RecBCD pathway of recombination describe this reaction in detail and depict the “Chi modulated DNA degradation” and “nick at Chi” models for the initiation of recombination shown in Fig 1A [2,3]. Following the formation of a D-loop through the strand exchange activity of RecA, Holliday junctions are formed, migrated and resolved by the RuvABC complex resulting in the formation of a structure resembling a replication fork. Subsequently, PriA is recruited to this fork-like structure, and is required to initiate a cascade of protein binding that ultimately results in the loading of the primary replicative helicase, DnaB, to the lagging-strand template [4]. DNA synthesis then proceeds to replace any genetic information lost at the site of the DSB (see [5] for a recent review). RecG has been a mysterious player in these reactions.

The observation that RecG not only plays a role in the RecBCD pathway of DSBR but also in the RecF and RecE pathways (activated in mutant strains of *E*. *coli*) suggests that, like RecA, it plays a fundamental role in DNA repair and acts on a DNA substrate that is common to different recombination pathways [6]. Indeed, its importance in DSBR has been confirmed using both cleavage of a chromosomal I-SceI target site with the I-SceI enzyme [7] and cleavage of a hairpin DNA structure by SbcCD nuclease [8,9]. Despite the early genetic evidence for a function common to three recombination pathways [6], many different roles for RecG have been proposed. These range from the migration of Holliday junctions to facilitate their resolution [7,10,11,12,13,14], the promotion and opposition of RecA strand exchange [15,16], the reversal of replication forks [17,18,19,20,21,22,23], the processing of flaps generated when DNA replication forks converge [24,25,26,27] and the stabilisation of D-loops [9]. Understanding the role of RecG has not been facilitated by the fact that the existence or identity of a eukaryotic homologue or functional orthologue has not been reported until recently [28]. If SMARCAL1 is indeed the human functional orthologue of RecG, there is hope that more light will be shed on the function of this important protein.

Purified RecG protein is a helicase that can bind and unwind synthetic model Holliday junctions [12]. *In vitro*, RecG efficiently catalyses the re-pairing of template strands in substrates mimicking replication forks, in a reaction termed replication fork reversal or replication fork regression [18,19,21,22,23]. Interestingly, this RecG promoted reaction occurs preferentially on substrates mimicking replication forks with a new strand annealed to the lagging-strand template [20,21]. RecG also efficiently reverses a replication fork blocked at a DNA lesion in an *in vitro* replication system where the DNA polymerase and replicative helicase are associated with the DNA [29]. These studies have led to a current view that an important biochemical action of RecG *in vitro* is replication fork reversal [30]. However in live cells there is a lack of evidence for RecG mediated fork reversal in several *in vivo* fork reversal reactions (e.g. [31]). Some indirect results imply that RecG might reverse replication forks following UV irradiation [19]. However following UV irradiation, the chromosome fragmentation by RuvABC-mediated cleavage of Holliday junctions present at reversed forks, which can be detected in a *recBC* mutant, is hardly affected by RecG [32]. This does not support even the view that RecG has a specific role in reversing forks following UV irradiation. The discordance between the substantial amount of evidence for RecG catalysed fork reversal *in vitro* and the small amount of evidence *in vivo* raises an interesting question: what is the substrate for RecG in live cells?

A clue as to the nature of the RecG substrate *in vivo* comes from the observation that a class of suppressors of the *recG* recombination deficient phenotype carry mutations in the helicase domain of PriA [33]. This is consistent with an interaction between RecG and PriA during the processing of recombination intermediates. PriA is required for the re-start of replication forks, during chromosomal DNA replication, recombination and replicative transposition, via the loading of the DnaB helicase [4,34,35,36]. Both RecG and PriA are known to remodel replication fork substrates *in vitro*. RecG binds the parental double-stranded part of a replication fork and unwinds the new strands (see [30] for a recent review). It has a preference to unwind a model fork substrate with a 5’ new lagging-strand at the fork over a substrate with a 3’ new leading-strand at the fork [20,21]. RecG unwinds the 5’ new lagging-strand and pairs it to the 3’ new leading-strand to generate a reversed fork [18,19,21,22,23,29]. However, in a coupled reaction where RecG and PriA are both present, RecG unwinds the 5’ new lagging-strand until a recessed 3’ new leading-strand end is brought to the branch point of the fork whereupon PriA binds in a configuration that does not lead to unwinding of parental template strands by the PriA helicase or continued unwinding by RecG [37]. A replication fork with a 3’ end at the branch point is a favoured substrate for PriA binding through the combined action of its N-terminal 3’ end binding domain (3’DB), a parental-strand binding winged helix domain (WH) and the helicase domains (HD1 and HD2) thought to contact the lagging-strand [38]. The biochemical literature supports the idea most clearly presented by Masai and colleagues [35] that RecG remodels replication forks to permit the 3’ end binding mode of PriA at a stalled fork or D-loop promoting the hand-off reaction to DnaB via PriB, DnaT and DnaC [39,40,41]. In the absence of a 3’ new leading-strand at the fork, PriA alone cannot be stabilised in the configuration in which its helicase is inactive for unwinding the parental duplex [35]. Instead, PriA moves from 3’ to 5’ on the leading-strand template to unwind the parental duplex and on the lagging-strand template to unwind the 5’ new lagging strand [35].

We show here that in the absence of RecG, abnormal DNA synthesis proceeds outwards and away from a specific site of attempted DSBR. Also, we show that in the absence of RecG attempted DSBR occurs at sites known to block DNA replication forks. Furthermore, we demonstrate that the DNA loss associated with the unwinding of joint molecules observed in the absence of both RecG and RuvAB requires PriA helicase activity. These results have led us to conclude that *in vivo* RecG plays a critical role in directing DNA synthesis at D-loops through its remodelling of the DNA to promote the correct binding of PriA. In turn, this has led us to reconsider the RecBCD recombination pathway in bacteria and to propose a mechanism in which the presence of a 5’ terminal DNA strand at a D-loop plays a more prominent role than generally envisaged.

## Results

### Chromosomal marker frequency analysis (MFA) following induction of a DSB in the absence of RecG

**Fig 2:**
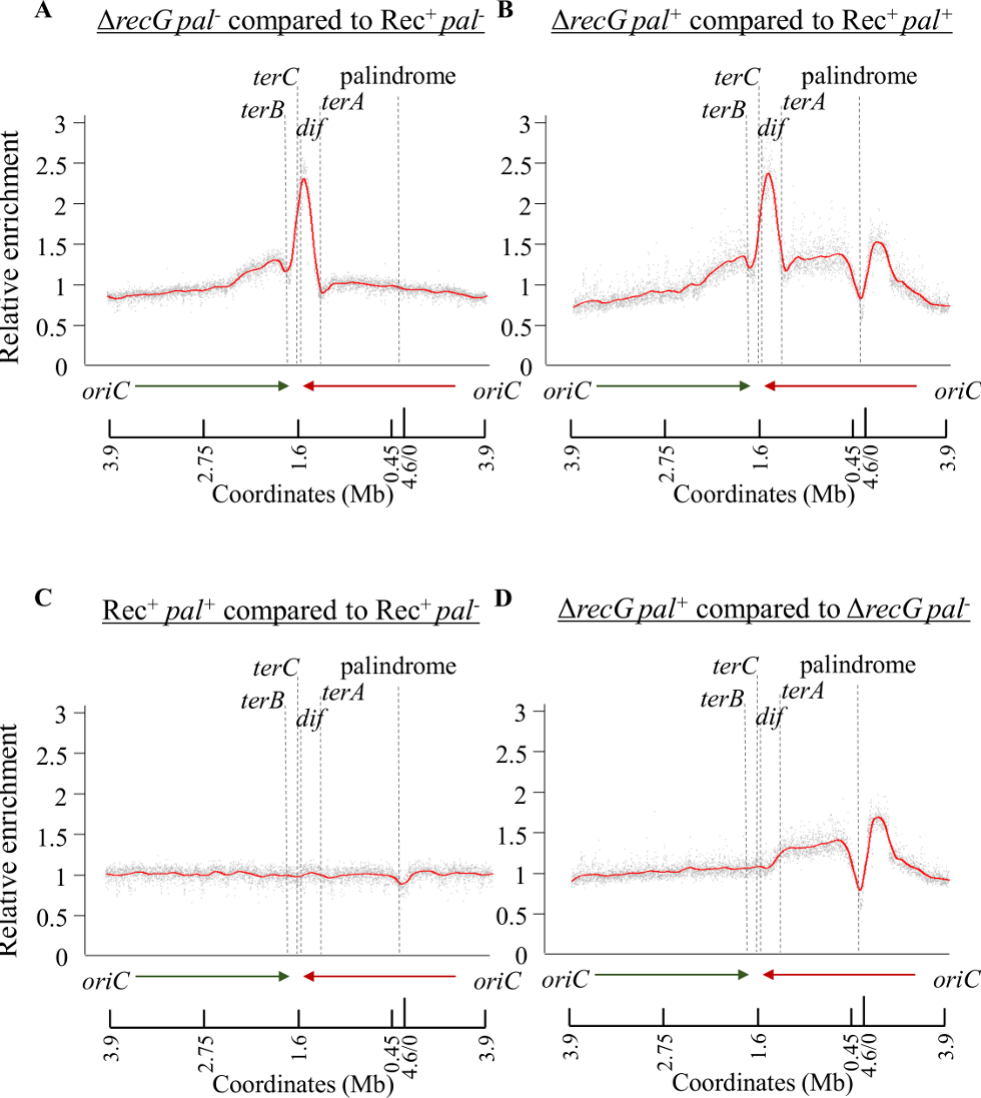
MFA profiles of Δ*recG* mutants and Rec^+^ strains of *E*. *coli* as a consequence of attempted DSBR at the *lacZ* locus. The ratio of the normalized DNA copy number (or “relative enrichment”) of uniquely mapped sequence reads from exponentially growing cultures of the strains of interest are plotted along the *y*-axis against replichore-formatted genomic coordinates along the *x*-axis. The average relative enrichment of DNA in a Δ*recG* mutant to a Rec^+^ strain is shown in the absence (A) or in the presence of an induced break at the palindrome (C), between Rec^+^ strains and Δ*recG* mutants in the presence and in the absence of an induced break at the palindrome (C-D). The relative positions of the replication termination sites (*terB*, *terC* and *terA*), *dif* site and the location of palindrome are shown for each plot. The data are the averages of the two biological replicates shown individually in supporting Information S1 Fig and S2 Fig. Strains used were DL4184 (Rec^+^ Pal^+^), DL4201 (Rec^+^ Pal^-^), DL4311 (Δ*recG* Pal^+^), and DL4312 (Δ*recG* Pal^-^).

We have used MFA by next generation genomic DNA sequencing to determine the DNA abundance profile in a *recG* deletion mutant following attempted DSBR at the site of an interrupted 246 bp palindrome (Pal^+^) in the *lacZ* gene of the *E*. *coli* chromosome (Fig 1B), following expression of the hairpin endonuclease SbcCD [8]. In the absence of a DSB at *lacZ*, the MFA pattern observed in a Δ*recG* mutant was as previously published [27,42]. An excess of DNA reads was detected in the region of the chromosome between the unidirectional termination sites, *terA* and *terB* (S1 Fig and S2 Fig). Normalisation of the number of mapped sequencing reads in a Δ*recG* mutant to the number of mapped reads in a Rec^+^ strain clearly revealed the excess of reads in the terminus region of the Δ*recG* mutant (Fig 2A). In the strain undergoing DSBR at the palindrome in *lacZ*, a similar pattern as in the strain that was not attempting DSBR was observed in the terminus region. However, there was also a loss of reads in the immediate vicinity of the DSB in *lacZ* followed by an excess of reads on both sides of this DSB (Fig 2B, S1 Fig and S2 Fig). The effect of attempted DSBR in the *lacZ* region is clearly visible (Fig 2D) when normalising the Δ*recG* Pal^+^ dataset (induced DSBR, in the presence of a 246 bp palindrome at *lacZ*) to the Δ*recG* dataset (no induced DSBR). Extra DNA accumulates on both sides of the DSBR site in a Δ*recG* mutant. This extra DNA, which is not observed in a Rec^+^ strain (Fig 2C), extends back towards the origin for about 300 kb and towards the terminus for about 1 Mb. It has previously been demonstrated that UV irradiated *recG* mutant cells undergo excess DNA replication that is not associated with initiation of DNA replication from the origin (*oriC*) [25,26]. Our work now shows that this abnormal DNA synthesis occurs on both sides of a site-specific DSBR event directly linking the location of DNA synthesis to the location of DSBR. In order to confirm that there is increased divergent DNA replication from the site of attempted DSBR in *lacZ* in the abnormal direction towards the origin, we inserted an ectopic *terB* site 50 kb origin-proximal of the palindrome in the orientation predicted to block replication forks progressing back towards the origin. We detect a 9-fold increase in replication fork blockage at this ectopic *terB* site in a *recG* mutant over Rec^+^ under conditions of DSBR at *lacZ* (S3 Fig) consistent with an increased level of divergent DNA replication in the *recG* mutant.

### In the absence of RecG, attempted DSBR occurs at sites of replication fork arrest

**Fig 3:**
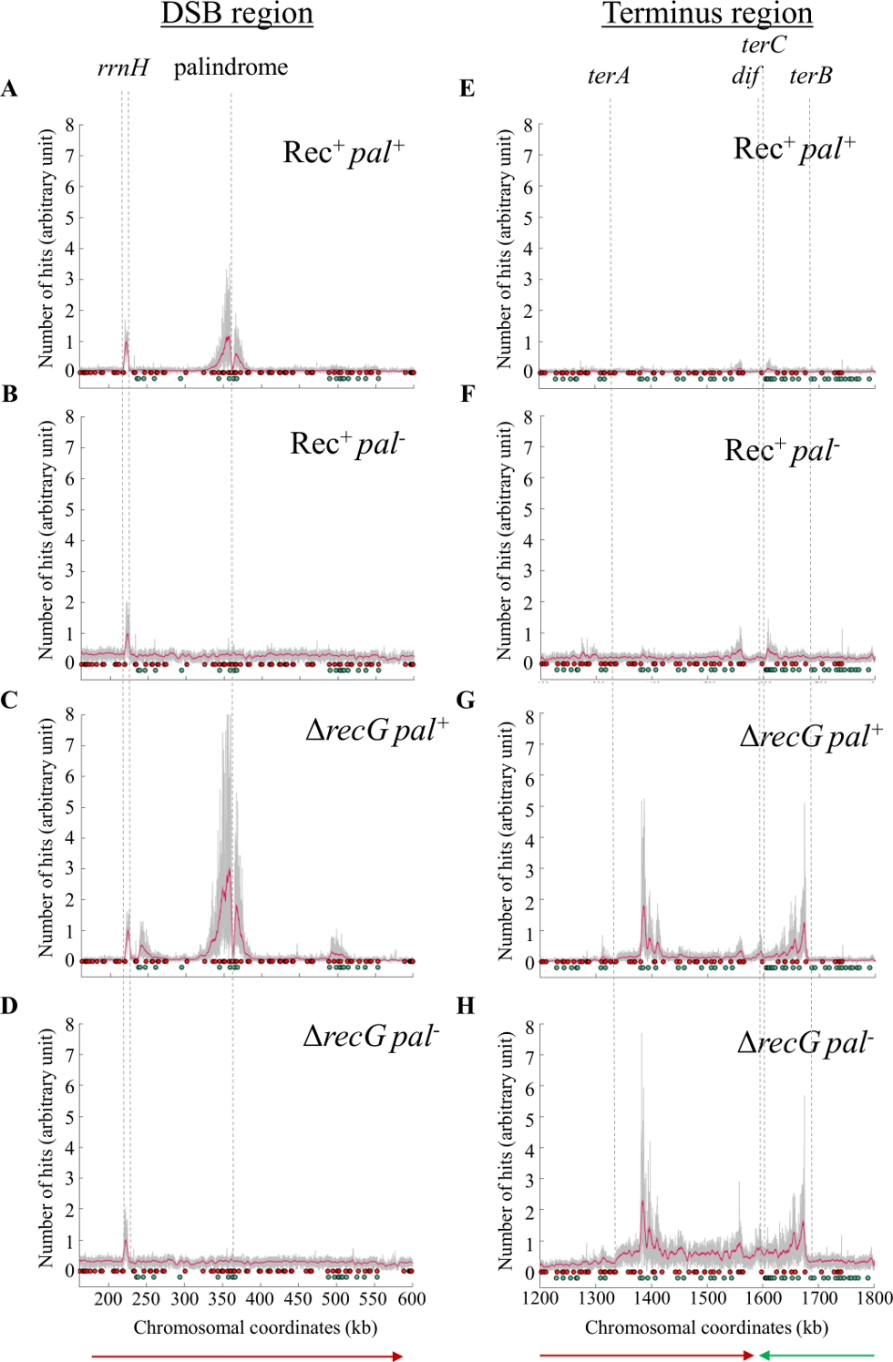
Relative RecA ChIP-seq reads in the *lacZ* DSBR region (A-D) and terminus region (E-H) of the chromosome. The raw data are shown in grey and smoothed data are shown in red. The smoothed data were plotted using a moving average filter with a 4 kb window. Red and green circles indicate Chi sites. Red Chi sites interact with RecBCD enzymes moving from right to left and green Chi sites interact with RecBCD enzymes moving from left to right. The closest Chi sites on either side of the DSB in *lacZ* were triple Chi arrays at 1.5kb from the palindrome, which have been used previously [9]. The positions of the *rrnH* operon, the palindrome at which a DSB is induced, termination sites (*terA*, *terB* and *terC*) and the site of resolution of chromosome dimers (*dif*) are all indicated. The direction of replication is indicated by green and red arrows for the left and the right replichore, respectively. A. and E. Rec^+^ Pal^+^; B. and F. Rec^+^ Pal^-^; C. and G. Δ*recG* Pal^+^; D. and H. Δ*recG* Pal^-^. Strains used were DL4184 (Rec^+^ Pal^+^), DL4201 (Rec^+^ Pal^-^), DL4311 (Δ*recG* Pal^+^), and DL4312 (Δ*recG* Pal^-^).

We have previously developed a method for visualising attempted DSBR that relies on chromatin immunoprecipitation of RecA cross-linked to DNA, followed by whole genome sequencing (RecA ChIP-seq; [43]). RecA is bound to DNA at sites of attempted DSBR following its loading at Chi sites by RecBCD. The shape of the RecA binding profile is distinctive. Binding rises sharply to a maximum value close to the position of a correctly oriented Chi site and then decreases with a slow exponential decay. This binding profile coupled to the locations and orientations of the Chi sites can be used to identify the region of the chromosome in which a DSB has been generated. These characteristics can also be used to distinguish between one-ended and two-ended breaks and to determine the directionality of a one-ended break. As can be seen in Fig 3A–3D, attempted DSBR in the presence and absence of RecG occurs at the site of palindrome cleavage in the *lacZ* gene. As expected from the results obtained in a Rec^+^ strain [43], RecA enrichment was observed on both sides of the break consistent with two-ended DSBR. In addition, three sites of attempted one-ended DSBR were specifically observed in the absence of RecG. The first of these was at a Chi site oriented appropriately if a replication fork proceeding from the site of the initial DSB in *lacZ* towards the origin of chromosomal replication generated a double-strand end at the closest ribosomal RNA operon (*rrnH*), 120 kb on the origin-proximal side of the DSB (Fig 3C). Because this replication fork would be proceeding in the reverse direction to normal chromosomal replication, it would have encountered the *rrnH* operon as it moved in the opposite direction to its transcription. Replication-transcription collisions of this kind are known to result in blocking of replication forks [44,45,46,47,48] and can generate one-ended DSBs [31]. It is worth noting that the *rrnH* operon itself is recognised by RecA but this recognition is independent of DSBR and independent of RecG. Furthermore, it bears no hallmarks of DSBR such as correlation with Chi sites or an asymmetric distribution (see [43] for further details of recombination independent RecA binding to rRNA genes). In a Δ*recG* mutant, RecA binding was also detected approximately 100 kb origin-distal to the DSB in *lacZ* in a Δ*recG* mutant (Fig 3C). This peak (which can also be detected at a low level in the Rec^+^ data) most likely corresponds to the origin-distal end of the DSB at *lacZ* being processed at a long distance. The elevated processing at a distance in a Δ*recG* mutant may be caused by unwinding of joint molecules followed by re-invasion downstream of the first Chi array or simply from RecBCD enzymes that had failed to recognise the first Chi array. The second and third sites of Δ*recG* specific attempted DSBR (Fig 3G and 3H) were located at positions of correctly oriented Chi sites for DSBs generated at the replication termination sites *terA* and *terB* [47,48]. Again these events were one-ended, consistent with replication fork processing, and were oriented appropriately for replication forks proceeding outward (from terminus towards origin) and being blocked at the termination sites. These sites of one-ended DSBR were also the boundaries of the extra terminal DNA replication that has been detected in Δ*recG* mutants by MFA (Fig 2 and S1 Fig) and [27,42].

### PriA helicase is responsible for the unwinding of joint molecules in the absence of RecG and RuvAB

We have shown previously that intermediates of DSBR are lost in a Δ*recG* Δ*ruvAB* double mutant [9] and have hypothesised that the branch migration activities of RecG and RuvAB stabilise joint molecules. Since RuvAB is a complex known to branch migrate Holliday junctions and to facilitate their resolution by cleavage in the presence of RuvC (see [5]), we considered it likely that the stabilising activity of RuvAB is mediated by branch migration of Holliday junctions. This was confirmed by the observation that 4-way junctions accumulated in a Δ*ruvAB* mutant [9]. However, the branch migration activity of RecG implicated in stabilising the joint molecules was less clear. The fact that 4-way junctions accumulated in the presence of RecG in a Δ*ruvAB* mutant indicated that they were not migrated away from the region of joint molecule formation by RecG. Instead, this suggested that RecG might stabilise joint molecules by remodelling the nascent fork end of the D-loop to promote DNA synthesis from the invading 3’ end.

**Fig 4:**
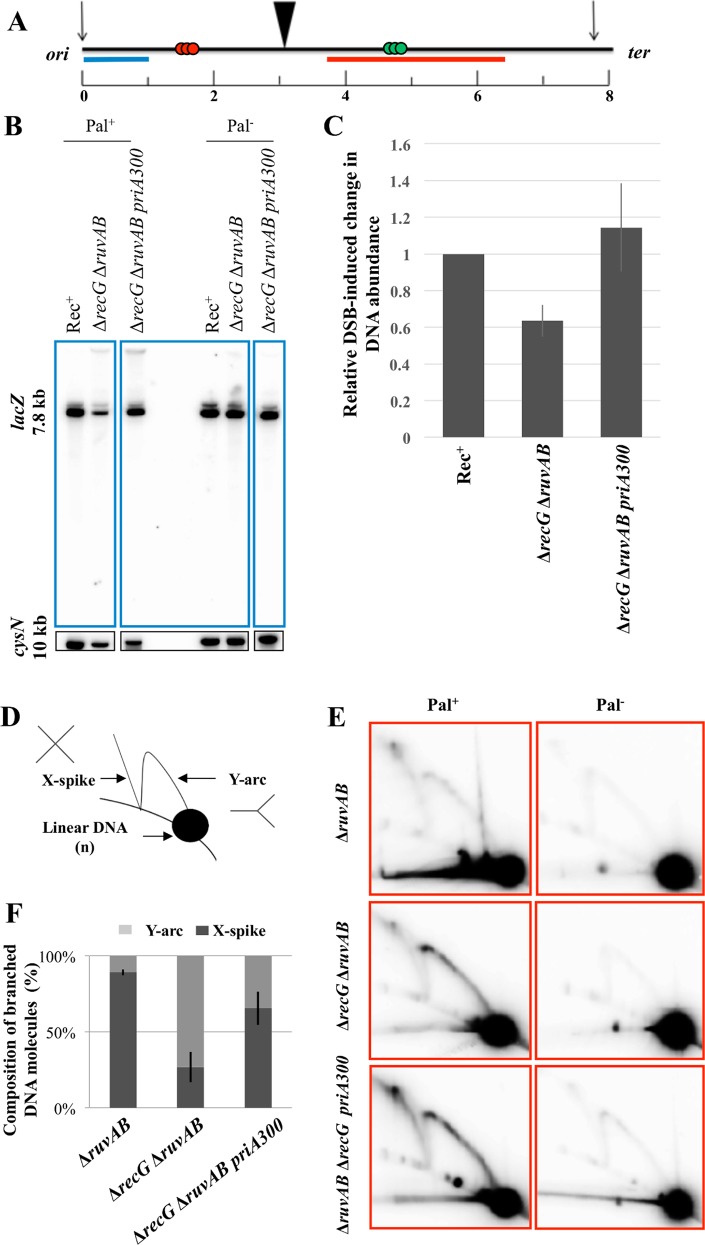
The *priA300* mutation suppresses the loss of DNA around a DSB in the absence of RecG and RuvAB. A. NdeI digestion map of the region surrounding the palindrome locus. NdeI cutting sites and the distance between them are marked with black vertical arrows and numbers (in kb), respectively. The palindrome is indicated by a black triangle, Chi arrays by three coloured circles, the *lacZ* probe by a blue line and the *lacZ*.*distal* probe by a red line. B. Southern blot of a 1% agarose gel probed with a *lacZ* fragment (top) and a cysN control fragment (bottom). Strains used were DL4184 (Rec^+^ Pal^+^), DL4260 (Δ*ruvAB* Δ*recG* Pal^+^), DL5610 (Δ*ruvAB* Δ*recG priA300* Pal^+^), DL4201 (Rec^+^ Pal^-^), DL4313 (Δ*ruvAB* Δ*recG* Pal^-^) and DL5611 (Δ*ruvAB* Δ*recG priA300* Pal^-^). C. Quantification of the total amount of DNA at and around the break site. These values were first normalised to the values for the *cysN* control fragment. Then these ratios for the Pal^+^ strains were normalised to their Pal^-^ controls. Finally, these ratios were normalised to the Rec+ ratio that was set to the value of 1. Error bars represent the standard error of the mean where n = 3. D. Schematic representation of the migration patterns of different species of branched DNA when separated on a two-dimensional native-native agarose gel. E. Two-dimensional native-native agarose gel electrophoresis. The DNA was detected using the *lacZ*.*distal* probe. Strains used were DL4243 (Δ*ruvAB* Pal^+^), DL4257 (Δ*ruvAB* Pal^-^), DL4260 (Δ*recG* Δ*ruvAB* Pal^+^), DL4313 (Δ*recG* Δ*ruvAB* Pal^-^), DL5610 (Δ*recG* Δ*ruvAB priA300* Pal^+^) and DL5611 (Δ*recG* Δ*ruvAB priA300* Pal^-^). F. Quantification of the DNA in the Y-arc and the X-spike normalised against the total branched DNA. Error bars represent the standard error of the mean where n = 3.

We have now tested whether the helicase activity of PriA is responsible for the DNA loss associated with destabilising joint molecules in the absence of RecG and RuvAB. The loss of DNA following induction of DSBR at *lacZ* was quantified by agarose gel electrophoresis and Southern hybridisation. The recovery of the 7.8 kb NdeI DNA fragment containing the DSB site in *lacZ* (Fig 4A) was compared to the recovery of the 10 kb NdeI *cysN* control fragment situated on the opposite side of the chromosome. As can be seen in Fig 4B and 4C, 40% of the DNA undergoing DSBR in a Δ*recG* Δ*ruvAB* mutant was lost from the *lacZ* region. This loss was prevented in a Δ*recG* Δ*ruvAB priA300* mutant, in which the helicase activity of PriA is inactivated by the K230R mutation [49]. The nature of the intermediates accumulated in a Δ*recG* Δ*ruvAB priA300* mutant was investigated by two-dimensional native-native agarose gel electrophoresis. In the absence of RecG and RuvAB, the *priA300* mutation increased the recovery of X-spike intermediates in the 7.8 kb NdeI fragment containing the DSB site, consistent with the accumulation of 4-way junctions (Fig 4E and 4F and S4 Fig). Our data suggest that the helicase activity of PriA is responsible for the unwinding of D-loops in the absence of the stabilising activities of RecG and RuvAB. Since the *priA300* mutation also suppresses the recombination deficiency of a *recG* mutant [50], we argue that it is RecG that prevents the unwinding activity of PriA helicase, suggesting that RecG is operating to facilitate the correct binding of PriA for DNA synthesis rather than D-loop unwinding.

## Discussion

In this work, we have made three principal observations pertaining to DSBR that is attempted in the absence of RecG. First, divergent replication occurs on both sides of the DSB (Fig 2). Second, stalled replication forks are processed to generate double-strand ends (at an *rrnH* operon, where collision between transcription and a divergent replication fork is expected, and at replication termination sites *terA* and *terB*) (Fig 3). Third, the helicase activity of PriA unwinds joint molecules in the absence of both RecG and RuvAB (Fig 4). We propose that RecG directs DNA synthesis at sites of DSBR and that this is mediated via the correct binding of the PriA. This proposal builds on the demonstration that RecG determines the correct binding of PriA *in vitro* [35,37] and reconciles a large body of literature describing the biochemical and genetic properties of both RecG and PriA.

### RecG directs DNA synthesis at sites of DSBR

**Fig 5:**
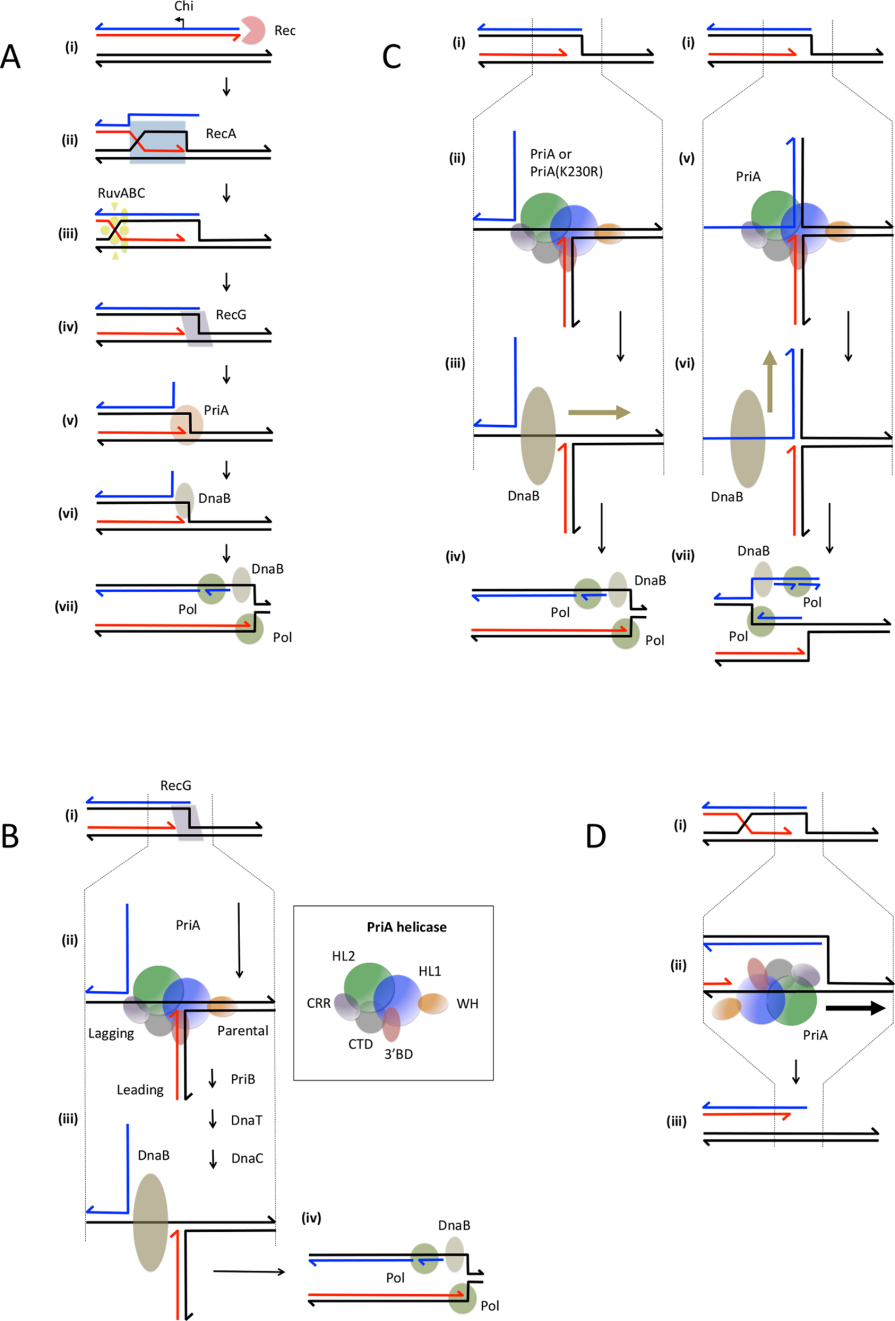
Model depicting the proposed action of RecG and PriA in the RecBCD recombination pathway. A. Revised model for the RecBCD recombination pathway. (i) The RecBCD enzyme recognises and binds to a DNA double-strand end. (ii) RecBCD generates a substrate with a 3’ end adjacent to a Chi site (shown as an arrow pointing in the direction of recombination stimulation) and a 5’ overhang. Continued unwinding by the RecBCD enzyme coupled to RecA loading onto the strand ending 3’ close to Chi allows the invasion of a target duplex and the formation of a D-loop. (iii) RuvAB binds to the Holliday junction end of the D-loop and migrates the Holliday junction away from the DSB end enlarging the D-loop. When a preferred recognition site for RuvC is encountered, the Holliday junction is resolved by cleavage and ligation. (iv) The RecG protein binds to the replication fork with a 5’ extended strand generated from the other end of the D-loop and unwinds the 5’ end while reannealing the parental DNA strands. (v) The action of RecG hands off the junction to PriA that binds in the correct manner to initiate the loading of DnaB. (vi) DnaB is loaded onto the lagging-strand template. (vii) DNA replication proceeds in the correct direction to restore the DNA lost in the region of the DSB. B. Hand-off between RecG and PriA ensures the correct loading of DnaB. The region between the dotted vertical lines is enlarged to show the binding of PriA. (i) RecG binds to a replication fork with an extended 5’ strand and unwinds this end while re-winding the parental template strands. (ii) This unwound fork is now in the right conformation to be bound by PriA in the orientation to load DnaB correctly onto the lagging-strand template. (iii) The hand-off reaction from PriA to PriB, to DnaT to DnaC to DnaB ensures that the replisome is reassembled. (iv) The replisome is loaded correctly to ensure the restoration of the DNA lost during the resection of the break. The box shows the domains of PriA as determined by X-ray crystallography [38]. The N terminus of the protein encodes the 3’ end-binding domain (3’BD–red). This is followed by a winged-helix domain proposed to interact with the parental DNA duplex (WH–orange). This is followed by two helicase lobes (HL1 –blue and HL2 –green). This is followed by a cysteine-rich region proposed to act as a wedge during helicase action (CRR–purple). Finally the protein is completed by a C-terminal domain that loops back round to the 3’BD (CTD–yellow). The *priA300* mutation is predicted to lie in the HL1 domain. C. Action of PriA in the absence of RecG. The region between the dotted vertical lines is enlarged to show the binding of PriA. (i) A replication fork substrate with an extended 5’ new end is available to bind PriA but is not specifically remodelled for this hand-off in the absence of RecG. (ii) and (v) Because the 3’ end is readily available, PriA remodels the fork to ensure that the 3’ end is bound by the 3’BD and the parental duplex is bound by the WH domain. In structure (ii), the PriA helicase domains (HL1 and HL2) bind correctly to the lagging-strand template and in structure (v) the helicase domains bind incorrectly to the new lagging-strand. The PriA(K230R) helicase (present in the *priA300* mutant) retains only the ability to bind correctly. (iii) and (vi) DnaB is loaded via the hand-off mechanism from PriA to PriB to DnaT to DnaC to DnaB. In (iii) DnaB is loaded correctly to the lagging-strand template and in (vi) DnaB is loaded incorrectly to the new lagging-strand. (iv) and (vii) A replication fork is reassembled. In (iv) the replication fork is assembled in the correct orientation to restore the DNA lost in the early stages of recombination at the site of the DSB. In (vii) the replication fork is assembled in the incorrect orientation and replicates the DNA flanking the DSB site. The direction of translocation of DnaB is indicated by a tan arrow. D. Action of PriA in the absence of RecG and RuvAB. The region between the dotted lines is enlarged to show the binding of PriA. (i) A replication fork with an extended 5’ “new” end is available for binding by PriA. However, the Holliday junction associated with the fork is not resolved by RuvABC and the fork itself is not remodelled by RecG. (ii) PriA has difficulty to remodel the fork to allow binding in the 3’ end-binding mode because the presence of the Holliday junction interferes with the required movement of the arms of the fork. This results in a significant proportion of molecules being bound by PriA in its helicase mode where it unwinds the parental duplex arms. (iii) This causes over-winding of the parental arms of the fork and under-winding of the D-loop, resulting in its dissociation. The direction of translocation of PriA is indicated by a black arrow.

Previous work has demonstrated that an excess of *oriC*-independent DNA replication occurs in a *recG* mutant following UV irradiation [25]. We have shown that at an induced DSB in *lacZ*, and adjacent to sites of one-ended breaks in the terminus region, there is DNA over-replication that proceeds away from the direction of appropriate replication (the direction of reconstitution of a replication fork at a D-loop). This establishes that the over-replication observed following attempted DSBR in a Δ*recG* mutant is associated with the site of DSBR itself. Previous work has shown that the over-replication observed following UV irradiation of a *recG* mutant is suppressed in *priA* helicase mutants implicating PriA in the over-replication phenotype [25]. Given the biochemical evidence that RecG remodels the DNA at a replication fork for the appropriate binding of PriA [37], we have considered whether, in the absence of RecG, PriA might bind to direct DNA synthesis inappropriately. In order for PriA to load DnaB incorrectly at the site of a D-loop, we envisage that PriA would bind in its 3’ end recognition mode in an orientation appropriate for loading DnaB onto the strand ending 5’ at the D-loop. We only see this as possible if the strand ending 5’ at the D-loop extends further than the 3’ ended strand (Fig 5).

How far this 5’ strand extends back towards the DSB site requires further investigation as does the fate of the 3’ strand from the DSB site to Chi. One can envisage two general scenarios based upon the known biochemistry of RecBCD enzyme (see [2,3,5] for recent reviews) and the models presented in Fig 1A. In one scenario, degradation of the 3’ end from the DSB site to Chi occurs frequently and the 5’ strand is cleaved infrequently leading to a recessed 3’ end at Chi. Following Chi recognition, unwinding by RecBCD continues but, in the presence of RecA, the Chi-activated 5’-3’ nuclease is inhibited, retaining the extended 5’ end. This would require an extension of the “Chi modulated DNA degradation” model [2] (see Fig 1A). In this new scenario, RecA loading would inhibit 5’ end cleavage by RecBCD after Chi recognition. In an alternative scenario, DNA from the DSB to Chi is unwound and the 3’ end is cleaved at Chi while the 5’ end remains intact. Following Chi recognition and cutting, unwinding continues and RecA is loaded to the 3’ strand. In this scenario, the unwound 3’ strand from the DSB site to Chi is somehow prevented from annealing to the 5’ strand. This might be accomplished by cleavage of the 3’ or 5’ stands before Chi by unknown nucleases (e.g. ExoI or RecJ) or by the binding of SSB to both unwound strands. This would require an extension of the “nick at Chi” model [3] to explain the fate of unwound strands between the DSB site and Chi. Previous studies have demonstrated that SSB attenuates RecBCD nuclease action and inhibits reannealing of strands unwound by RecBCD [51,52,53,54]. These actions of SSB are likely to promote the persistence of a protruding 5’ single-stand provided the Chi-activated 5’-3’ nuclease of RecBCD is not operating (e.g. because of the ionic conditions or because of RecA loading).

Our model is summarised in Fig 5A. We envisage that RecBCD enables loading of RecA to a 3’ single-strand generated by unwinding beyond the cleaved Chi site and that a joint molecule is formed that retains a 5’ tail. RuvABC migrates and resolves the Holliday junction at one end of this joint molecule allowing the formation of a replication fork with an extended 5’ end. This is the preferred substrate for RecG [20,21]. RecG binds and unwinds the 5’ end while reannealing the parental template stands of the fork but hands off to PriA before unwinding of the 3’ end can occur [37], thus preventing fork reversal. In Fig 5B we show how PriA is expected to bind to permit the loading of DnaB to the lagging-strand template. In Fig 5C we compare the two possible binding modes of PriA to a substrate with a 5’ new strand at the fork in the absence of a hand-off reaction from RecG. It can be seen that a simple rotation of strands coupled to displacement of the 5’ end can lead to alternative 3’ end-binding modes that predict either loading of DnaB onto the lagging-strand template (correct loading) or onto the new lagging-strand (incorrect loading). Because the 3’ end is available and PriA can manipulate the junction both binding modes involve recognition of the 3’ end and lead to DnaB loading rather than helicase activity.

### In the absence of RecG, PriA helicase can unwind D-loops that have not been converted to replication forks by RuvABC

Joint molecules are formed through the action of RecBCD and RecA. We have previously proposed that in the absence of RuvAB and RecG these joint molecules are unstable because D-loops cannot be converted to replication forks by RuvABC action and because RecG is not present to carry out an unknown stabilising role [9]. We considered that this stabilising role could either be the migration of the Holliday junction away from the site of DSBR or the establishment of correct DNA synthesis from the site of the D-loop. Given the known suppression of the *recG* recombination defective phenotype by helicase mutants of PriA and our observation of inappropriate backward-directed DNA synthesis at sites of attempted DSBR in a Δ*recG* mutant we sought to test whether PriA helicase activity might unwind D-loops in the absence of RecG and RuvAB. Our data reveal that the helicase activity of PriA is indeed responsible for the DNA loss associated with destabilisation of joint molecules in a Δ*ruvAB* Δ*recG* mutant. Two possible modes of unwinding by PriA helicase that have been observed *in vitro* might be responsible for this. Unwinding of the 5’ end would directly unpair one of the D-loop double-strands, while unwinding the parental duplex strands would cause strand rotation that would unwind the D-loop (D-loop migration). We consider that the unwinding of the parental duplex and the consequent unwinding of the D-loop by strand rotation, required to minimise accumulation of positive supercoils (ahead of the D-loop) and negative supercoils (behind the D-loop) during its migration, is likely to be the critical activity of PriA helicase in this situation. This is because this action would result in ejection of both the 3’ and the 5’ ends from the D-loop, which would be needed to unwind the joint molecules. This action of PriA helicase requires an extended 5’ end at the replication fork side of the D-loop, to provide the single-stranded DNA region for PriA binding on the leading-strand template. This is consistent with our view that such an end is indeed present. We envisage that remodelling of the replication fork end of the D-loop is prevented in the absence of RuvAB by a persistent Holliday junction that tethers the two strands of the fork. This prevents the binding of PriA in the 3’ end-binding mode required for DnaB loading and leaves only the helicase mode of PriA binding available as shown in Fig 5D.

### The role of RecG in terminus over-replication

As seen previously in a *recG* mutant [27,42], we observe DNA over-replication in the terminus region of the chromosome between the sequences *terA* and *terB*. This over-replication is eliminated in helicase mutants of PriA [27]. We show here that terminus over-replication in the absence of RecG is not influenced by attempted DSBR at *lacZ* but is associated with attempted DSBR at *terA* and *terB* as revealed by RecA binding at the positions of the first correctly-oriented Chi sites adjacent to these *ter* sites. We therefore propose that this over-replication is caused by a similar reaction to the backward replication from D-loops that we envisage happening at the DSBR event in *lacZ*. Because the DSBs at *terA* and at *terB* are one-ended and outward-facing, they do not arise from replication fork collision in the centre of the terminus region as envisaged in the model proposed by Lloyd and colleagues [24,25,26,27,42]. Furthermore, our demonstration of backward-directed replication at a site of attempted DSBR in *lacZ* and of one-ended DSBR at *terA* and *terB* do not fit with the model of Gowrishakar [55] that does not envisage replication initiation in the terminus region.

**Fig 6:**
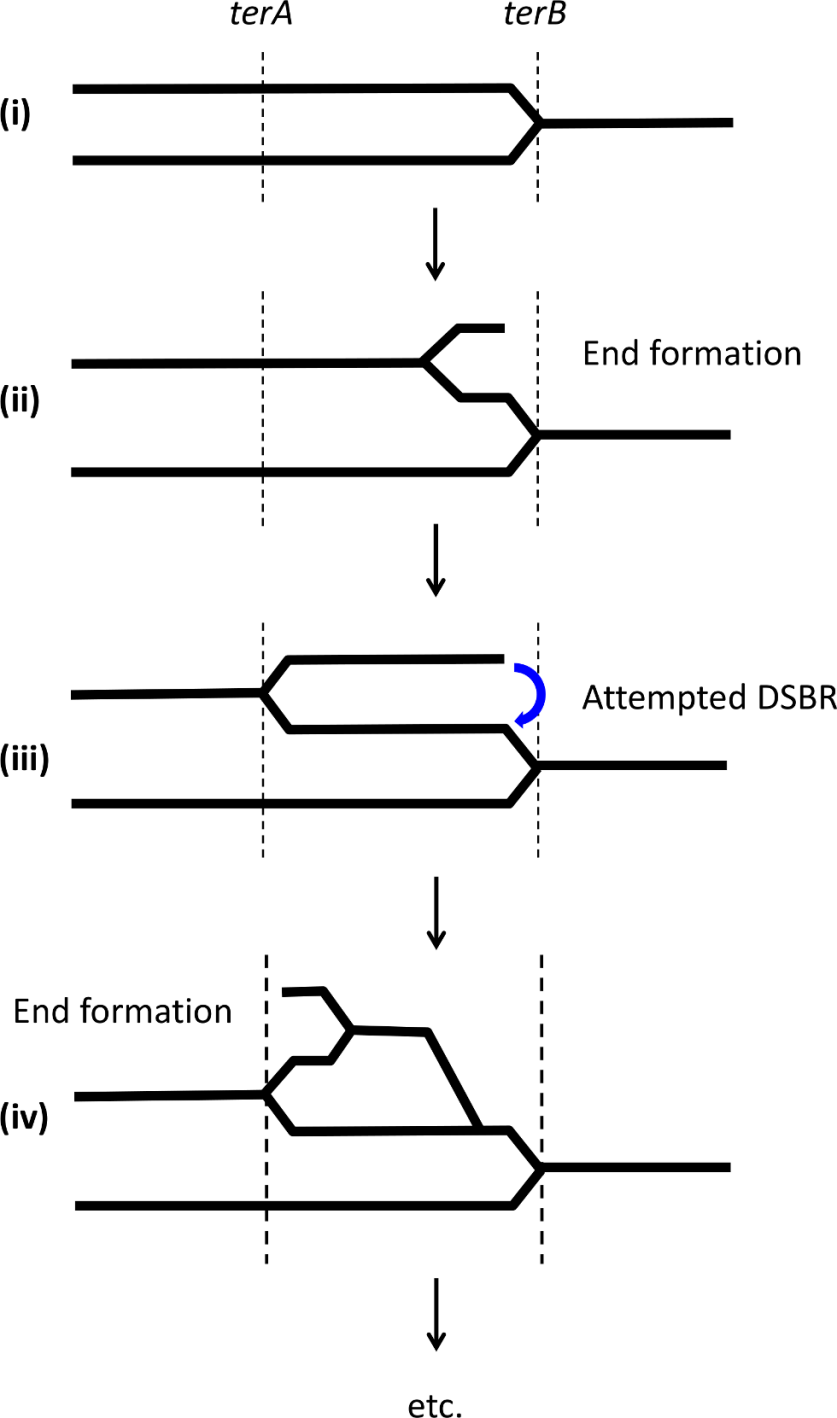
Model proposed for the over-replication of the terminus region between *terA* and *terB* in the absence of RecG. (i) A replication fork is shown having traversed the terminus region in the direction from *terA* to *terB* where it is arrested. (ii) In the absence of RecG, PriA binds incorrectly at *terB* and causes a replication fork to be assembled that moves in the reverse direction towards *terA*. Whether the arrested fork is originally broken and repair is attempted prior to the assembly of the backward-directed replication fork is unknown. Whether or not the fork is broken, a single DNA end is generated adjacent to *terB*. (iii) The backward directed replication fork is blocked at *terA* and recombination of the DNA end with the intact duplex is attempted. (iv) The same process of assembly of a backward directed replication fork is set up at *terA*, this time moving towards *terB*. This game of ping-pong between *terA* and *terB* continues indefinitely with a preference for attempted DSBR events close to the ter sites but also with more internal sites derived from the D-loops generated from attempted recombination events. Replication forks can either start the process by being blocked at *terB* (as shown in (i)) or at *terA*. The combination of all the events occurring in the population results in the accumulation of DNA observed in a Δ*recG* mutant between *terA* and *terB*.

A depiction of how we envisage terminus replication in the absence of RecG is shown in Fig 6. We propose that in the absence of RecG, a replication fork that has been blocked by collision with a Tus/*ter* complex is no longer protected from incorrect binding of PriA helicase. This results in the deposition of DnaB on the newly synthesised strand ending 5’ close to *ter* and the establishment of a fork that moves back across the terminus region until it is stopped by encounter with another *ter* site. At this point, another backward-directed replication fork can be assembled and replication can copy the same region again in the opposite direction. In the meantime the ends generated by backward-directed replication will attempt recombination and so create more forks that can set up more backward-directed replication as well as forks that will collide with the original *ter* sites. This cascade of replication in the absence of RecG explains the DNA over-replication of the terminus region. The initial formation of replication forks blocked at the *ter* sites in a Δ*recG* mutant is likely to be contributed to by stable DNA replication as suggested previously [55].

The precise molecular details of how PriA binds in the terminus region require further investigation. It is known that Tus protein blocks DNA synthesis initially leaving a recessed 5’ end of 50–100 nt [56]. It is possible that this is a poor substrate for the hand-off reaction from RecG to PriA but is converted to a good substrate via the action of 3’ to 5’ exonucleases, the absence of which can cause RecG independent replication in the terminus region [27,42]. Alternatively, Tus protein itself modifies the interaction of PriA with DNA in the absence of RecG.

### Conclusion

We have shown that in the absence of RecG attempted DSBR at either the site of an induced two-ended DSB in *lacZ*, or at a site in which a replication fork is predicted to collide with a transcription bubble (at the *rrnH* operon), or at sites in which replication forks are expected to collide with the Tus/ter complex at ter sites, abnormal backward-directed DNA synthesis is observed. Furthermore, we have shown that D-loops that have not been acted upon by RuvABC or RecG are unwound by the helicase activity of PriA. These results strongly suggest that RecG acts at the replication fork end of a D-loop and possibly at a stalled replication fork to direct the correct loading of the DnaB replicative helicase through the correct binding of PriA. This conclusion is supported by the biochemical evidence that the action of RecG allows PriA to associate with a synthetic replication fork substrate with a recessed 3’ end in its 3’ end-binding mode in which it can promote the further hand-off reaction to DnaB rather than acting as a helicase [37]. This new understanding of the role of RecG reconciles many roles previously proposed. The synergistic action of RecG and RuvAB is explained by alternative modes of stabilising D-loops. The apparent contradiction that RecG strongly promotes replication fork reversal *in vitro* whereas little evidence for this reaction has been obtained *in vivo* is explained by the hand-off reaction from RecG to PriA, which captures a key DNA structure and prevents fork reversal *in vivo*. The single situation in which fork reversal has been proposed to occur *in vivo* is following UV irradiation [19]. It is possible that the extent of damage overwhelms the ability of PriA to capture all the precursors to fork reversal. There is no longer any need to propose a role for RecG in the processing of flaps hypothesised to occur at sites of convergent replication forks [24,25,26,27] as the fork collision model is not supported by the outward facing one-ended attempted DSBR that we infer at *ter* sites in the absence of RecG. Our new understanding also explains why RecG has a preference for action at a replication fork substrate with an extended 5’ end. This is indeed the substrate that we hypothesise normally to be present in a D-loop since we propose that the extended 5’ end is required for the inappropriate binding of PriA (in its incorrect 3’ end-binding mode) in the absence of RecG. It is also the structure that we hypothesise to be required for the incorrect binding of PriA (in its helicase mode) in the absence of RuvAB and RecG.

According to this view, RecG may be considered an early participant in the hand-off reaction from PriA to DnaB, which is required for the re-start of replication during DSBR. This pathway may be considered to run from RecG to PriA to PriB to DnaT to DnaC to DnaB [39,57,58,59,60]. Given that a pathway of replication restart from a DSB has not yet been identified in eukaryotic cells it will be interesting to know whether the potential human functional orthologue of RecG (SMARCAL1) opens a window on this important reaction in higher organisms.

## Materials and Methods

### Strains and oligonucleotide sequences used

All strains and oligonucleotide sequences used are listed in supporting information S1 and S2 Tables (S1 Table: DNA oligonucleotide sequences used and S2 Table: Bacterial strains used).

### Plasmid construction

The plasmid pDL4922 (Cm^R^ Ts Suc^s^) was created in order to introduce a *terB* site (5’-AATAAGTATGTTGTAACTAAAGT-3’) site in between the pseudogenes *ykgM* and *eaeH* of the *E*. *coli* chromosome to pause counter clockwise replication forks specifically. Primer pairs used for the cross-over PCR on BW27784 genomic DNA were ykgMterB-F1 /R1 and ykgMterB-F2/R2. These primers permit the insertion of a *terB* site between the two homology arms. This fragment was cloned in pTOF24 using PstI and SalI restriction enzymes [61].

The plasmid pDL4947 (Cm^R^ Ts Suc^s^) was created in order to introduce the *priA300* mutation into the *priA* locus of the *E*. *coli* chromosome. The region was amplified from JJC1422 using *priA300*.F and *priA300*.R primers, digested using SalI and PstI and inserted into the temperature sensitive plasmid pTOF24.

### Induction of DSBs

Overnight cultures were grown in 5ml of LB medium. The following day, cultures were diluted to an OD_600nm_ of 0.02 and grown shaking at 37°C to an OD_600nm_ of 0.2. Cultures were then re-diluted to an OD_600nm_ of 0.02 and grown shaking at 37°C to an OD_600nm_ of 0.2. Expression from the P_*BAD*_*-sbcDC* construct was induced by the addition of 0.2% arabinose to the culture medium. Cultures were then incubated at 37°C for 1 hour before samples were isolated.

### Sample preparation for MFA by genomic DNA sequencing

DNA was isolated from cultures after 1 hour induction of *sbcDC* expression using the Promega Wizard^®^ Genomic DNA purification kit by following the manufacturer’s instructions. RNase treatment was carried out for 50 minutes and the DNA was re-hydrated overnight in TE (10 mM Tris (pH 7.4), 1 mM EDTA) at 4°C. To further eliminate potential RNA, 3 units of Riboshredder (RNase Blend) were added per sample according to the manufacturer’s instructions. Samples were purified by phenol/chloroform extraction and ethanol precipitation. The integrity of the DNA was verified by running the samples on a 0.8% agarose gel and the quantity of DNA was determined by Nanodrop analysis (Thermo Scientific) and by Qubit fluorometry (Life Technologies). Finally, construction of libraries and DNA sequencing was carried out on an Illumina HiSeq 2000 platform by Edinburgh Genomics, using the Illumina TruSeq DNA Sample Prep kit according to manufacturer’s instructions.

### MFA data analysis

Paired-end raw datasets from an Illumina HiSeq 2000 sequencing platform (obtained from Edinburgh Genomics) were mapped against the genomic sequence of the reference strain ‘BW27784’ using BWA sequence aligner (version 0.7.11) and subsequently analysed using SAMtools (version 1.2). ‘BW27784’ is a modified version of *E*. *coli* K12 MG1655 (NC000913.3) including all published differences between the strains [62,63]. Replication profiles of exponentially growing cultures were calculated by normalizing to the number of uniquely mapped sequence reads (to correct for differences in depth of sequencing) and then to the normalised reads of a non-replicating stationary-phase wild-type culture (a Rec^+^ strain without palindrome) to correct for differences in sequence-based recovery across the genome. An in-lab R-script (available on request) has been used to calculate the enrichment (normalised read depth) in 1 kb non-overlapping windows across the genome and a non-parametric smoothing method (LOESS, Local regression) has been applied to the data points of the replication profiles of each strain.

### ChIP sample preparation

All ChIP experiments were performed with cells grown in exponential growth phase. RecA-DNA interactions were chemically cross-linked with formaldehyde (Sigma-Aldrich, at a final concentration of 1%) for 10 minutes at 22.5°C. Crosslinking was quenched by the addition of 0.5 M glycine (Sigma-Aldrich). Cells were collected by centrifugation at 1,500 x g for 10 minutes and then washed three times in ice-cold 1X PBS. The pellet was then re-suspended in 250 μl ChIP buffer (200 mM Tris-HCl (pH 8.0), 600 mM NaCl 4% Triton X, Complete protease inhibitor cocktail EDTA-free (Roche)). Sonication of crosslinked samples was performed using the Diagenode Bioruptor at 30 seconds intervals for 10 minutes at high amplitude. After sonication, 350 μl of ChIP buffer was added to each sample, the samples were mixed by gentle pipetting and 100 μl of each lysate were removed and stored as ‘input’. Immunoprecipitation was performed overnight at 4°C using 1/100 anti-RecA antibody (Abcam, ab63797). Immunoprecipitated (IP) samples were then incubated with Protein G Dynabeads® (Life Technologies) for 2 hours with rotation at room temperature. All samples were washed three times with 1 X PBS + 0.02% Tween-20 before re-suspending the Protein G dynabeads in 200 μl of TE buffer + 1% SDS. 100 μl of TE buffer were added to the input samples and all samples were then incubated at 65°C for 10 hours to reverse the formaldehyde cross-links. DNA was isolated using the MinElute PCR purification kit (Qiagen) according to manufacturer’s instructions. DNA was eluted in 100 μl of TE buffer using a 2-step elution. Samples were stored at -20°C.

### ChIP library preparation for high-throughput sequencing

Input and ChIP samples were processed following NEB’s protocol from the NEBNext ChIP-Seq library preparation kit. Briefly, input and ChIP-enriched DNA were subjected to end repair to fill in ssDNA overhangs, remove 3’ phosphates and phosphorylate the 5’ ends of sheared DNA. Klenow exo- was used to adenylate the 3’ ends of the DNA and NEXTflex DNA barcodes (Bioo Scientific) were ligated using T4 DNA ligase. After each step, the DNA was purified using the Qiagen MinElute PCR purification kit according to the manufacturer’s instructions. After adaptor ligation, the adaptor-modified DNA fragments were enriched by PCR using primers corresponding to the beginning of each adaptor. Finally, agarose gel electrophoresis was used to size select adaptor-ligated DNA with an average size of approximately 275 bp. All samples were quantified on a Bioanalyzer (Agilent) before being sequenced on the Illumina® HiSeq 2000 by BGI International.

### ChIP-Seq data analysis

50 bp single-end reads were mapped to the *E*. *coli* K12 ‘BW27784’ genome using Novoalign version 2.07 (www.novocraft.com). Novoalign uses the Needleman-Wunsch algorithm to determine the optimal alignment of reads. Before mapping, the 3’ adaptor sequences were removed using fastx_clipper and the data collapsed using fastx_collapser to remove identical sequence reads (http://hannonlab.cshl.edu/fastx_toolkit/index.html). Sequences were mapped with default parameters, allowing for a maximum of one mismatch per read. In order to report reads that have multiple alignment loci we specified the–r parameter as “Random”. PyReadCounters was used to calculate the overlap between aligned reads and *E*. *coli* genomic features [64]. The distribution of reads along the *E*. *coli* genome was visualized using the Integrated Genome Browser [65]. Full details of all scripts are available upon request.

The raw data are shown in grey and smoothed data are shown in red. The smoothed data were plotted using a moving average filter with a 4 kb window. The data have been normalised relative to the peak of RecA ChIP observed at the *rrnH* locus. This peak of RecA ChIP is independent of induced DSBR at *lacZ*, is independent of the *recG* genotype and does not have the characteristics of DSBR (it is not correlated with the positions of Chi sites and the binding is uniform across the gene). Whether or not this binding is of biological interest or represents a ChIP artefact remains to be determined. However, it usefully provides a way of approximately normalising reads between experiments. This normalisation cannot be considered absolute as this peak may itself be influenced by unknown factors that differ between experiments. We are therefore careful not to infer absolute levels of RecA binding between experiments.

### DNA analysis by gel electrophoresis

Methods were adapted from [9,66]

#### (a) Isolation of chromosomal DNA in agarose plugs

After 1 hour of *sbcCD* induction, cells were harvested at 4°C and washed 3 times in TEN buffer (50 mM Tris, 50 mM EDTA, 100 mM NaCl, pH 8.0). Cells were re-suspended in TEN buffer to an OD_600nm_ of 6 or 80 for conventional agarose gels or native/native two-dimensional gels, respectively. The cells were then mixed with an equal volume of 2% (for conventional gels) or 0.8% (for two dimensional gels) of low melting point agarose (Invitrogen) prepared in TEN buffer and equilibrated to 37°C. The mix was poured into plug moulds (BioRad) and allowed to set for 1 hour. Plugs were treated in NDS solution (0.5 M EDTA, 10 mM Tris, 0.55 M NaOH, 36.8 mM lauroyl sarcosine; pH 8.0) supplemented with 1 mg/ml of proteinase K (Roche) for an overnight shaking at 37°C. Fresh NDS + proteinase K were added for a second overnight incubation. Following this treatment, plugs were stored at 4°C in fresh NDS. Before digestion of the DNA, a plug was washed in 1 x restriction buffer 6 times, replacing the buffer every hour. The plug was then placed in fresh 1 x restriction buffer, supplemented with the restriction enzyme and incubated rocking at 37°C overnight.

#### (b) Agarose gel electrophoresis

An agarose plug containing digested DNA was run on a 1% (w/v) agarose gel in 0.5 x TBE (44.5 mM Tris-borate, 1mM EDTA) at 2 V/cm for 12 hours at 4°C. The DNA was transferred to a positively charged nylon membrane (GE heathcare hybond+) by Southern blotting and cross-linked using UV-light.

#### (c) Native/native two dimensional agarose gel electrophoresis

An agarose plug containing digested DNA was run in the first dimension on a 0.4% (w/v) agarose gel in 1 x TBE (89 mM Tris-borate, 2 mM EDTA) at 1 V/cm for either 24 (for 4 kb fragment) or 36 hours (for 8 kb fragment) at 4°C. The lane was cut out, rotated 90°, and set in the second dimension agarose (1% in 1 x TBE supplemented with 0.3 μg/ml of ethidium bromide). The second dimension was run at 6 V/cm for either 10 (for 4 kb fragment) or 14 hours (for 8 kb fragment) at 4°C. The DNA was transferred to a positively charged nylon membrane (GE heathcare hybond+) by Southern blotting and cross-linked using UV-light.

#### (d) Radioactive detection of DNA

DNA was detected using ^32^P α-dATP incorporated into a PCR fragment (using Stratagene Prime-It II random primer labelling kit). Probes were hybridised to membranes overnight at 65°C in 10 ml of Church-Gilbert buffer (7% SDS, 0.5 M NaH_2_PO_4_, 1 mM EDTA, 1% BSA). Membranes were washed for 15 minutes at 60°C in 2X SSC (1X SSC: 0.15 M NaCl, 0.015 M Na-citrate) supplemented with 0.1% SDS and then 30 minutes in 0.5 x SSC supplemented with 0.1% SDS. Labelled membranes were exposed to GE healthcare storage phosphor screens and scanned using a Molecular Dynamics Storm 860 phosphorImager scanner. Images were quantified using GE healthcare ImageQuant TL.

#### (e) Analyses of loss of DNA following Southern blotting

To quantify the loss of DNA, the data obtained from *lacZ* probing were normalised to the data obtained from the probing of the *cysN* control fragment, located on the opposite side of the chromosome. The background signal was subtracted and the data were normalised to the no palindrome control.

## Supporting Information

S1 Table DNA oligonucleotides used. (DOCX)

S2 Table Bacterial strains used. (DOCX)

S1 Fig Replication profiles of individual biological replicates of Δ*recG* mutants and Rec^+^ strains. Replication profiles of exponentially growing cultures of Rec^+^ strains with (A) or without (B) the palindrome and a Δ*recG* mutant with (C) or without (D) the palindrome are shown. In each graph, log_2_ of the normalized copy number of uniquely mapped sequence reads (log_2_ DNA abundance) is plotted along the *y*-axis against replichore-formatted genomic coordinates along the *x*-axis. The directions of chromosomal replication are depicted with green and red arrows to indicate the left and right replichores, respectively. The relative positions of the replication termination sites (*terB terC* and *terA*), the *dif* site and the palindrome are shown for each plot.
(TIFF)

S2 Fig Comparative analysis of replication profiles between biological replicates of *recG* mutants and Rec^+^ strains of *Escherichia coli*. Replication profiles across the genome of growing cultures of Δ*recG* mutants with and without the palindrome are shown in (A). The same has been shown for Δ*recG* mutants and RecG^+^ strains with the palindrome in (B), and for Δ*recG* mutants and Rec^+^ strains without a palindrome in (C). In all cultures the expression of SbcCD was induced for one hour prior to isolation of the DNA. In each graph, log_2_ of the normalized copy number of uniquely mapped sequence reads (log_2_ DNA abundance) is plotted along the *y*-axis against replichore-formatted genomic coordinates along the *x*-axis. The continuous and dotted lines represent biological replicates of the experiment. The directions of chromosomal replication are depicted either with a green arrow to indicate left replichore or a red arrow to indicate the right replichore. The relative positions of the replication termination sites (*terB*, *terC* and *terA*), the *dif* site and the location of the palindrome are shown for each plot. This analysis was carried out because of the notable difference in enrichment of mapped sequence reads on the two sides of the induced DSB in *lacZ* in the Δ*recG* mutant. All other duplicates correspond closely across their genome as do the two biological replicates with an induced DSB in *lacZ* in the *recG* mutant in the left replichore and the terminus region. The basis for the notable differences on the two sides of the induced DSB in the Δ*recG* mutant requires further investigation. Nevertheless, because both replicates show enrichment of sequence reads on both sides of the induced DSB we conclude that this particular behaviour is reproducible and we have presented the average relative enrichment in Fig 2.
(TIFF)

S3 Fig Divergent replication forks are elevated in a Δ*recG* mutant subjected to DSBs. **A.** PvuII digestion map of the region 50 kb upstream of the palindrome locus. PvuII cutting sites and the distance between them are marked with black vertical arrows and numbers (in kb), respectively. The *terB* site and the *ykgM*.*3* probe are marked by a green shape and a blue line, respectively. **B.** 2-D native-native agarose gel electrophoresis. The DNA was detected using the *ykgM*.*3* probe. Some partial digestion products are visible on the gels. Strains used were DL5096 (Rec^+^ *lacZ*::*246 ykgM-terB*), DL5097 (Rec^+^ *lacZ*^*+*^ *ykgM-terB*), DL6033 (Δ*recG lacZ*::*246 ykgM-terB*), and DL6034 (Δ*recG lacZ*^*+*^ *ykgM-terB*). **C.** Quantification of the paused forks relative to the linear DNA. Proportion of signal at the *terB* over linear DNA was calculated. Then, the data obtained from palindrome containing strains were normalised to the data obtained from no palindrome control. Finally, the signal obtained from Rec^+^ strain were subtracted from Δ*recG* sample. Error bars represent the standard error of the mean where n = 3.
(TIFF)

S4 Fig Further quantification of X-spike and Y-arc intermediates. Quantification of X-spike and Y-arc intermediates compared to linear DNA in the Δ*ruvAB*, Δ*recG* Δ*ruvAB*, and Δ*ruvAB* Δ*recG priA300* strains subjected to DSBs (data from Fig 4E). Error bars represent the standard error of the mean where n = 3.
(TIFF)
