# Supplementary material for: RecG Directs DNA Synthesis during Double-Strand Break Repair
Source: PLoS Genet. 2016 Feb 12;12(2):e1005799. doi: 10.1371/journal.pgen.1005799 (PMC4752480; doi:10.1371/journal.pgen.1005799)
Supplement: S1 Table — (DOCX) [file pgen.1005799.s001.docx]

**Table S1: DNA oligonucleotide sequences used**

| **Name** | **Sequence (5' to 3')** | **Purpose** |
| --- | --- | --- |
| *cynN.*F | GTCGTTATGCAGCGATGAGA | To make the *cysN* probe |
| *cynN.*R | GCGGAAAGCATCCACATTAT |  |
| *lacZ*p.F | TAGCGGCTGATGTTGAACTG | To make the *lacZ* probe |
| *lacZ*p.R | ATGAACGGTCTGGTCTTTGC |  |
| *lacZ.*distal.p.F | ATCGTCGTATCCCACTACCG | To make the *lacZ*.distal probe |
| *lacZ.*distal.p.R | TTTCCATGCGAGGTTAAAGG |  |
| *priA300*.F | AAAAACTGCAGTTTCCTGCTTCTTCGCTTTC | To construct pDL4947 |
| *priA300*.R | AAAAAGTCGACATCTCTCGCTCATCGCAAAG |  |
| *ykgMterB.*F1 | AAAAACTGCAGTCGTTCATGAGAAGCATAACGTA | To construct pDL4922 |
| *ykgMterB.*R1 | AATAAGTATGTTGTAACTAAAGTGCCACCTAATATATCCAGTG |  |
| *ykgMterB.*F2 | ACTTTAGTTACAACATACTTATTGGTGCACTTTCTTACATAAAG |  |
| *ykgMterB.*R2 | AAAAAGTCGA**C**TTACGTGTCGCATCGCTATC |  |
