# Supplementary material for: RecG Directs DNA Synthesis during Double-Strand Break Repair
Source: PLoS Genet. 2016 Feb 12;12(2):e1005799. doi: 10.1371/journal.pgen.1005799 (PMC4752480; doi:10.1371/journal.pgen.1005799)
Supplement: S2 Table — (DOCX) [file pgen.1005799.s002.docx]

**Table S2: Bacterial strains used**

| **Strain** | **Genotype** | **Source** |
| --- | --- | --- |
| BW27784 | Δ(*araD-araB*)*567* Δ(*araH-araF*)*570*(::*FRT*) Δ*araEp-532*::*FRT φP_cp18_araE533* Δ(*rhaD-rhaB*)*568 hsdR514* Δ*lacZ478*(::*rrnB-3*) | [[1](#_ENREF_1)] |
| JJC1422 | DM4100 *priA300* *pflD*::miniTn*10*Kan^R^ | [[2](#_ENREF_2)] |
| N3793 | AB1157 Δ*recG263*::Kan^R^ | [[3](#_ENREF_3)] |
| DL4184 | BW27784 *lacZ*::χχχ *mhpR*::χχχ *proA*::*ISceI_cs_ tsx*::*ISceI_cs_* P*_BAD_-sbcDC lacZ:: pal246 cynX*::Gm^R^ *lacI^q^ lacZχ****^-^**** | [[4](#_ENREF_4)] |
| DL4201 | BW27784 *lacZ*::χχχ *mhpR*::χχχ *proA*::*ISceI_cs_ tsx*::*ISceI_cs_* P*_BAD_-sbcDC lacZ^+^ cynX*::Gm^R^ *lacI^q^ lacZχ****^-^**** | [[4](#_ENREF_4)] |
| DL4243 | DL4184 Δ*ruvAB* | [[4](#_ENREF_4)] |
| DL4260 | DL4243 Δ*recG263*::Kan^R^ | [[4](#_ENREF_4)] |
| DL4311 | DL4184 Δ*recG263*::Kan^R^ | [[4](#_ENREF_4)] |
| DL4312 | DL4201 Δ*recG263*::Kan^R^ | [[4](#_ENREF_4)] |
| DL4313 | DL4201 Δ*recG263*::Kan^R^ Δ*ruvAB* | [[4](#_ENREF_4)] |
| DL5096 | DL4184 *ykgM*-*terB* | This work |
| DL5097 | DL4201 *ykgM*-*terB* | This work |
| DL5423 | DL4184 *priA300* | This work |
| DL5424 | DL4201 *priA300* | This work |
| DL5610 | DL5423 Δ*recG263*::Kan^R^ Δ*ruvAB* | This work |
| DL5611 | DL5424 Δ*recG263*::Kan^R^ Δ*ruvAB* | This work |
| DL6033 | DL5096 Δ*recG263*-Kan^R^ | This work |
| DL6034 | DL5097 Δ*recG263*-Kan^R^ | This work |

* Endogenous Chi site in *lacZ* has been deleted.

**References :**

1. Khlebnikov A, Datsenko KA, Skaug T, Wanner BL, Keasling JD (2001) Homogeneous expression of the P(BAD) promoter in Escherichia coli by constitutive expression of the low-affinity high-capacity AraE transporter. Microbiology 147: 3241-3247.

2. Flores MJ, Ehrlich SD, Michel B (2002) Primosome assembly requirement for replication restart in the Escherichia coli holDG10 replication mutant. Mol Microbiol 44: 783-792.

3. Al-Deib AA, Mahdi AA, Lloyd RG (1996) Modulation of recombination and DNA repair by the RecG and PriA helicases of Escherichia coli K-12. J Bacteriol 178: 6782-6789.

4. Mawer JS, Leach DR (2014) Branch migration prevents DNA loss during double-strand break repair. PLoS Genet 10: e1004485.
