# Supplementary figures and images for: RecG Directs DNA Synthesis during Double-Strand Break Repair
Source: PLoS Genet. 2016 Feb 12;12(2):e1005799. doi: 10.1371/journal.pgen.1005799 (PMC4752480; doi:10.1371/journal.pgen.1005799)

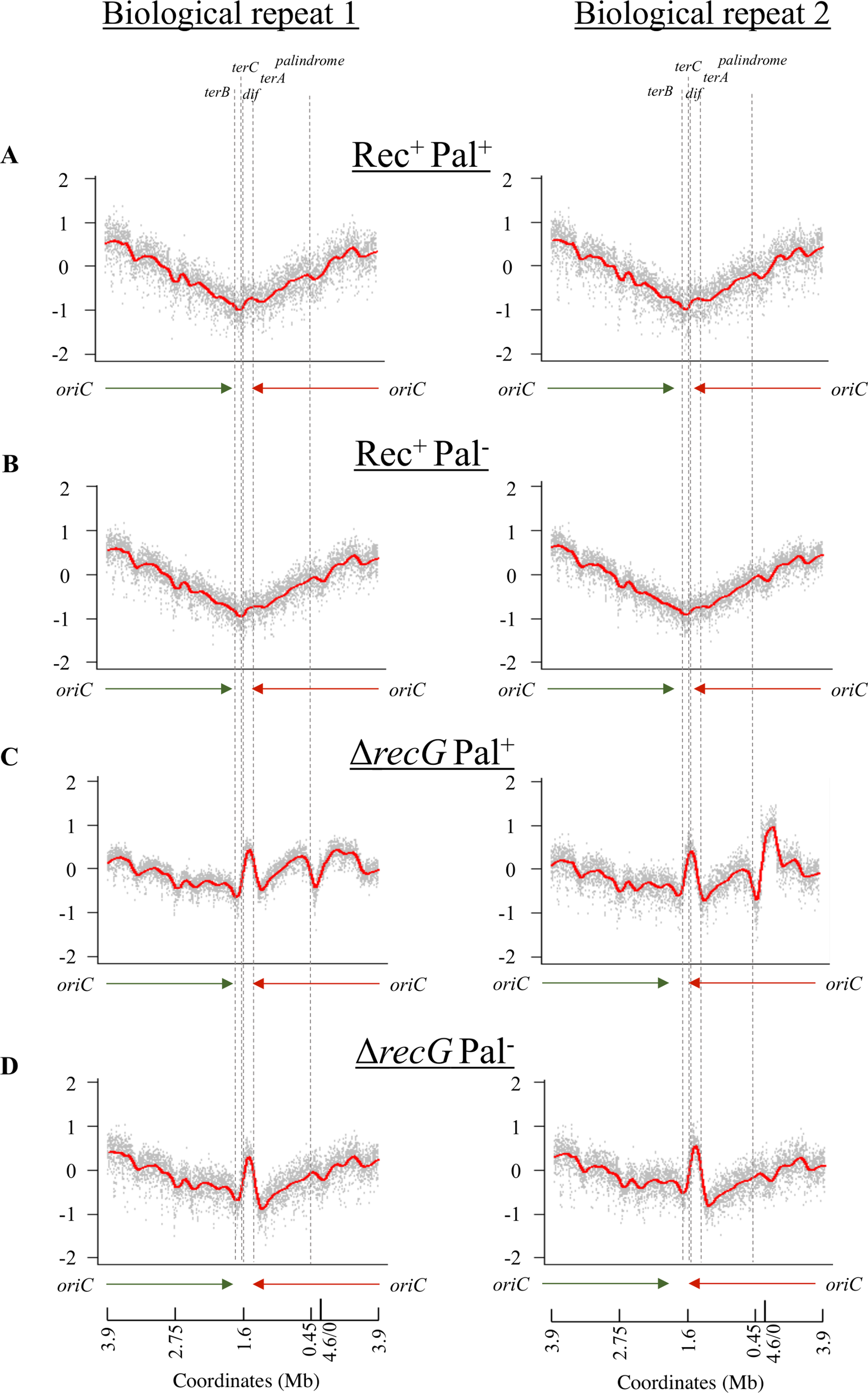

Supplement: S1 Fig — Replication profiles of exponentially growing cultures of Rec+ strains with (A) or without (B) the palindrome and a ΔrecG mutant with (C) or without (D) the palindrome are shown. In each graph, log2 of the normalized copy number of uniquely mapped sequence reads (log2 DNA abundance) is plotted along the y-axis against replichore-formatted genomic coordinates along the x-axis. The directions of chromosomal replication are depicted with green and red arrows to indicate the left and right replichores, respectively. The relative positions of the replication termination sites (terB terC and terA), the dif site and the palindrome are shown for each plot. (TIFF) [file pgen.1005799.s003.tiff]

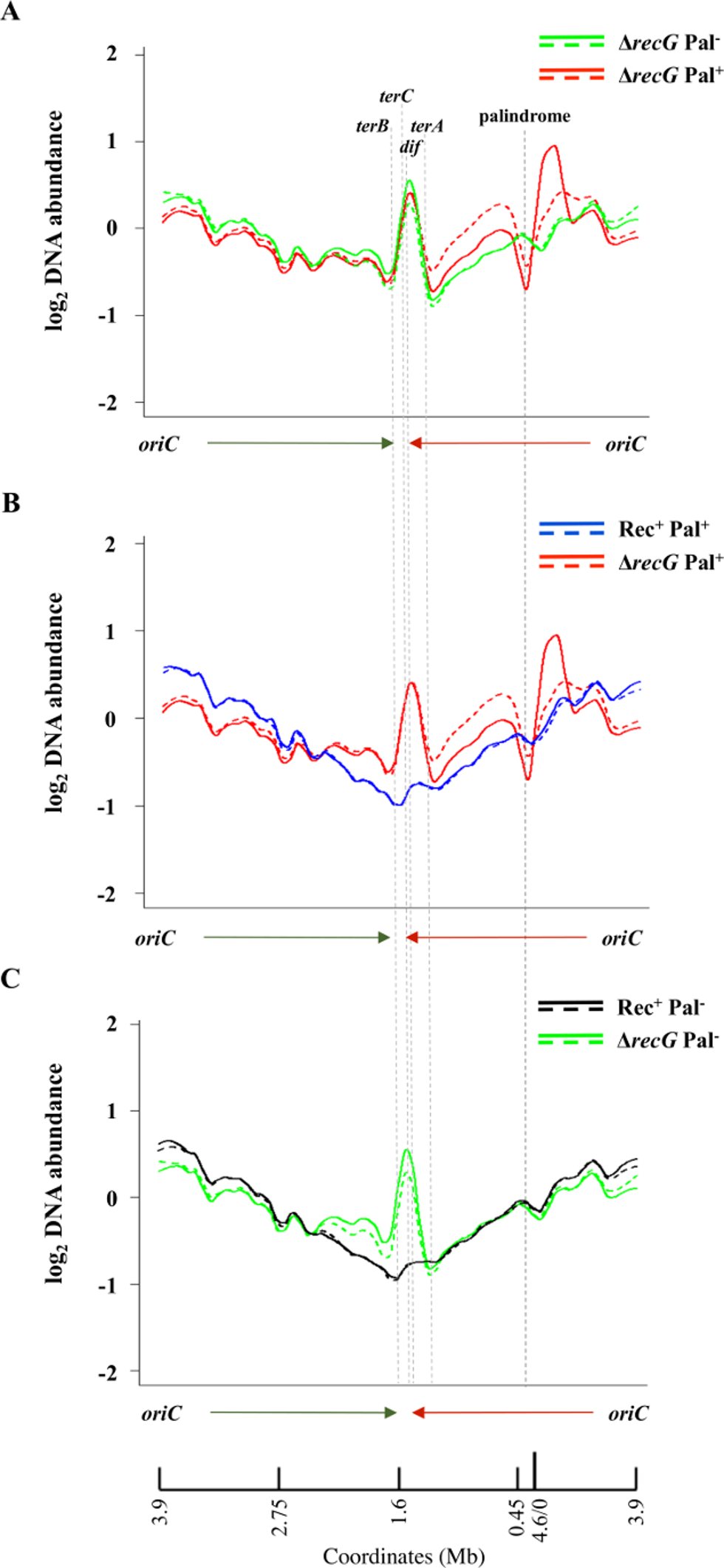

Supplement: S2 Fig — Replication profiles across the genome of growing cultures of ΔrecG mutants with and without the palindrome are shown in (A). The same has been shown for ΔrecG mutants and RecG+ strains with the palindrome in (B), and for ΔrecG mutants and Rec+ strains without a palindrome in (C). In all cultures the expression of SbcCD was induced for one hour prior to isolation of the DNA. In each graph, log2 of the normalized copy number of uniquely mapped sequence reads (log2 DNA abundance) is plotted along the y-axis against replichore-formatted genomic coordinates along the x-axis. The continuous and dotted lines represent biological replicates of the experiment. The directions of chromosomal replication are depicted either with a green arrow to indicate left replichore or a red arrow to indicate the right replichore. The relative positions of the replication termination sites (terB, terC and terA), the dif site and the location of the palindrome are shown for each plot. This analysis was carried out because of the notable difference in enrichment of mapped sequence reads on the two sides of the induced DSB in lacZ in the ΔrecG mutant. All other duplicates correspond closely across their genome as do the two biological replicates with an induced DSB in lacZ in the recG mutant in the left replichore and the terminus region. The basis for the notable differences on the two sides of the induced DSB in the ΔrecG mutant requires further investigation. Nevertheless, because both replicates show enrichment of sequence reads on both sides of the induced DSB we conclude that this particular behaviour is reproducible and we have presented the average relative enrichment in Fig 2. (TIFF) [file pgen.1005799.s004.tiff]

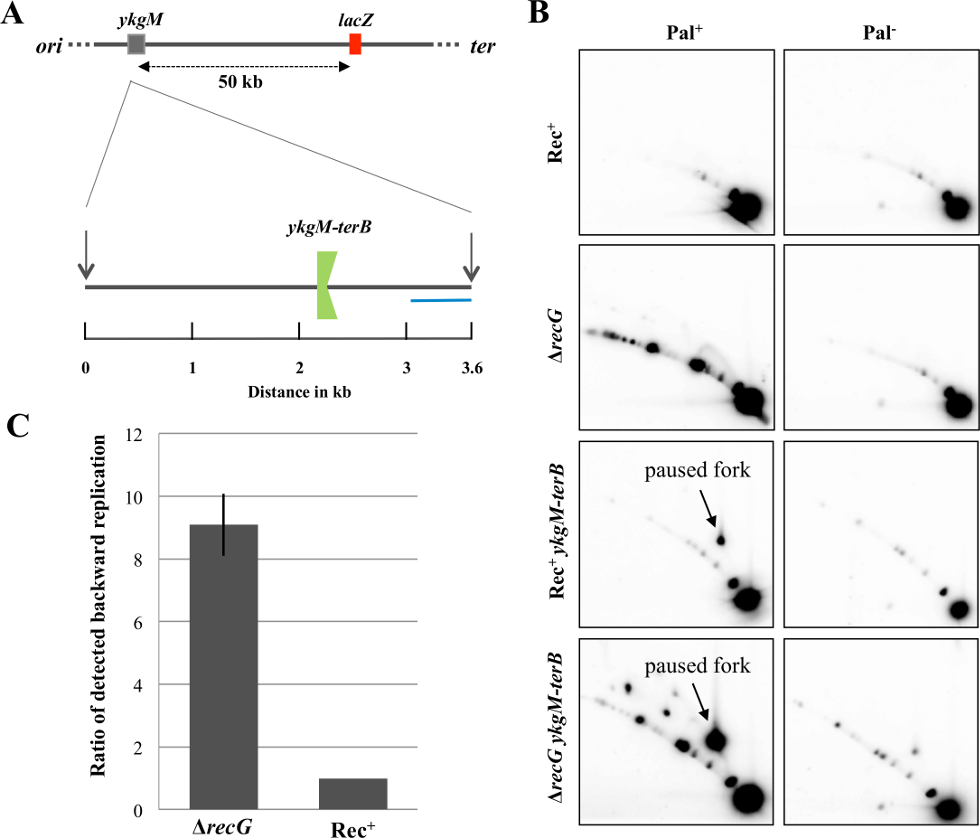

Supplement: S3 Fig — A. PvuII digestion map of the region 50 kb upstream of the palindrome locus. PvuII cutting sites and the distance between them are marked with black vertical arrows and numbers (in kb), respectively. The terB site and the ykgM.3 probe are marked by a green shape and a blue line, respectively. B. 2-D native-native agarose gel electrophoresis. The DNA was detected using the ykgM.3 probe. Some partial digestion products are visible on the gels. Strains used were DL5096 (Rec+ lacZ::246 ykgM-terB), DL5097 (Rec+ lacZ+ ykgM-terB), DL6033 (ΔrecG lacZ::246 ykgM-terB), and DL6034 (ΔrecG lacZ+ ykgM-terB). C. Quantification of the paused forks relative to the linear DNA. Proportion of signal at the terB over linear DNA was calculated. Then, the data obtained from palindrome containing strains were normalised to the data obtained from no palindrome control. Finally, the signal obtained from Rec+ strain were subtracted from ΔrecG sample. Error bars represent the standard error of the mean where n = 3. (TIFF) [file pgen.1005799.s005.tiff]

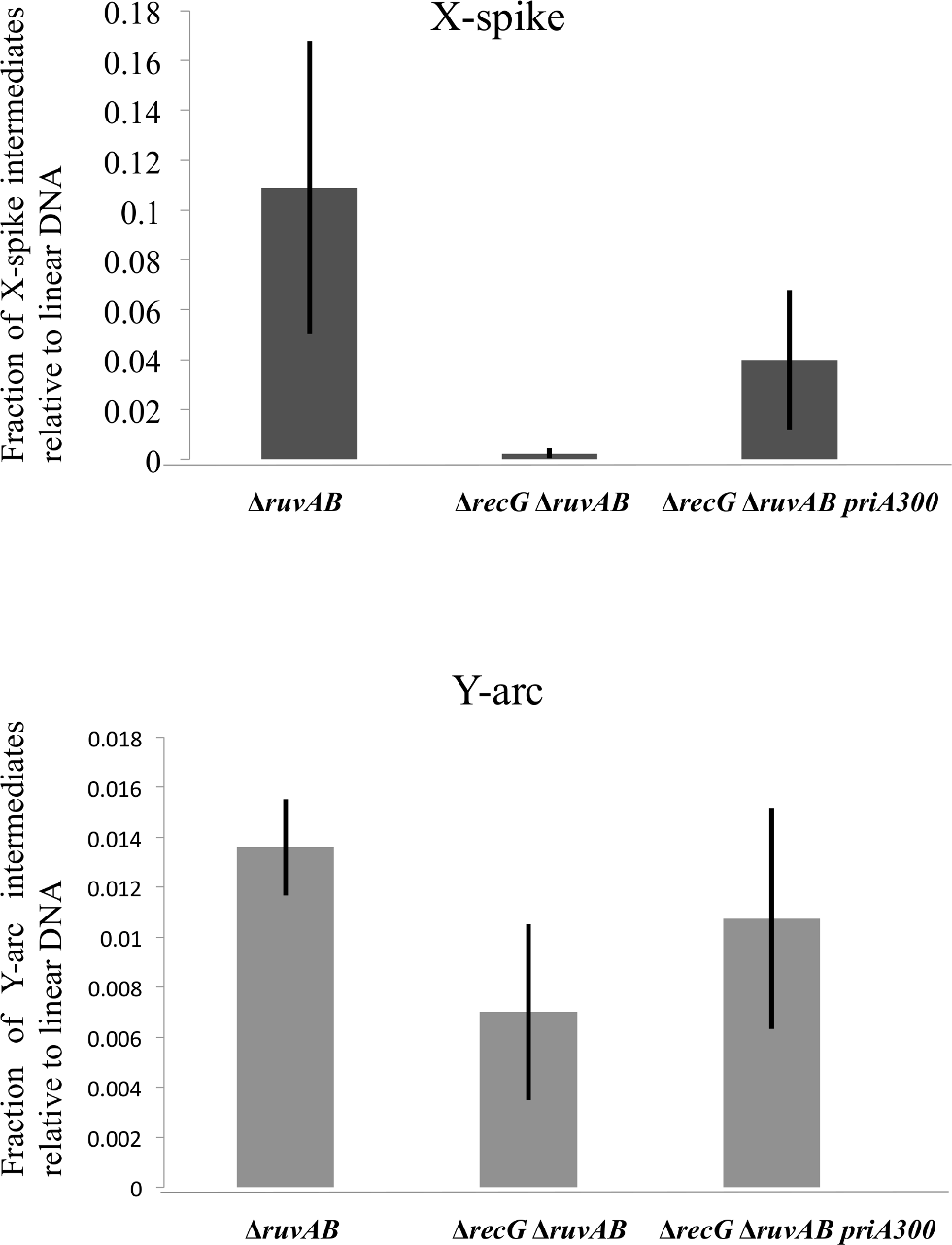

Supplement: S4 Fig — Quantification of X-spike and Y-arc intermediates compared to linear DNA in the ΔruvAB, ΔrecG ΔruvAB, and ΔruvAB ΔrecG priA300 strains subjected to DSBs (data from Fig 4E). Error bars represent the standard error of the mean where n = 3. (TIFF) [file pgen.1005799.s006.tiff]
